# Evaluation of size-dependent uptake, transport and cytotoxicity of polystyrene microplastic in a blood-brain barrier (BBB) model

**DOI:** 10.1186/s40580-024-00448-z

**Published:** 2024-10-15

**Authors:** Yeongseon Cho, Eun U Seo, Kyeong Seob Hwang, Hyelim Kim, Jonghoon Choi, Hong Nam Kim

**Affiliations:** 1 Brain Science Institute, Korea Institute of Science and Technology (KIST), Seoul, 02792 Republic of Korea; 2 School of Integrative Engineering, Chung-Ang University, Seoul, 06974 Republic of Korea; 3 Division of Bio-Medical Science and Technology, KIST School, University of Science and Technology (UST), Seoul, 02792 Republic of Korea; 4 Department of Biotechnology, Yonsei University, Seoul, 03722 Republic of Korea; 5 University of Science and Technology, Seoul, 02792 Republic of Korea; 6 School of Mechanical Engineering, Yonsei University, Seoul, 03722 Republic of Korea; 7 Yonsei-Korea Institute of Science and Technology Convergence Research Institute, Yonsei University, Seoul, 03722 Republic of Korea

**Keywords:** Microplastic, Blood-brain barrier, Uptake, Toxicity, Polystyrene

## Abstract

Microplastics, particularly those in the micrometer scale, have been shown to enter the human body through ingestion, inhalation, and dermal contact. Recent research indicates that microplastics can potentially impact the central nervous system (CNS) by crossing the blood-brain barrier (BBB). However, the exact mechanisms of their transport, uptake, and subsequent toxicity at BBB remain unclear. In this study, we evaluated the size-dependent uptake and cytotoxicity of polystyrene microparticles using an engineered BBB model. Our findings demonstrate that 0.2 μm polystyrene microparticles exhibit significantly higher uptake and transendothelial transport compared to 1.0 μm polystyrene microparticles, leading to increased permeability and cellular damage. After 24 h of exposure, permeability increased by 15.6-fold for the 0.2 μm particles and 2-fold for the 1.0 μm particles compared to the control. After 72 h of exposure, permeability further increased by 27.3-fold for the 0.2 μm particles and a 4.5-fold for the 1.0 μm particles compared to the control. Notably, microplastics administration following TNF-α treatment resulted in enhanced absorption and greater BBB damage compared to non-stimulated conditions. Additionally, the size-dependent toxicity observed differently between 2D cultured cells and 3D BBB models, highlighting the importance of testing models in evaluating environmental toxicity.

**Graphical Abstract:**

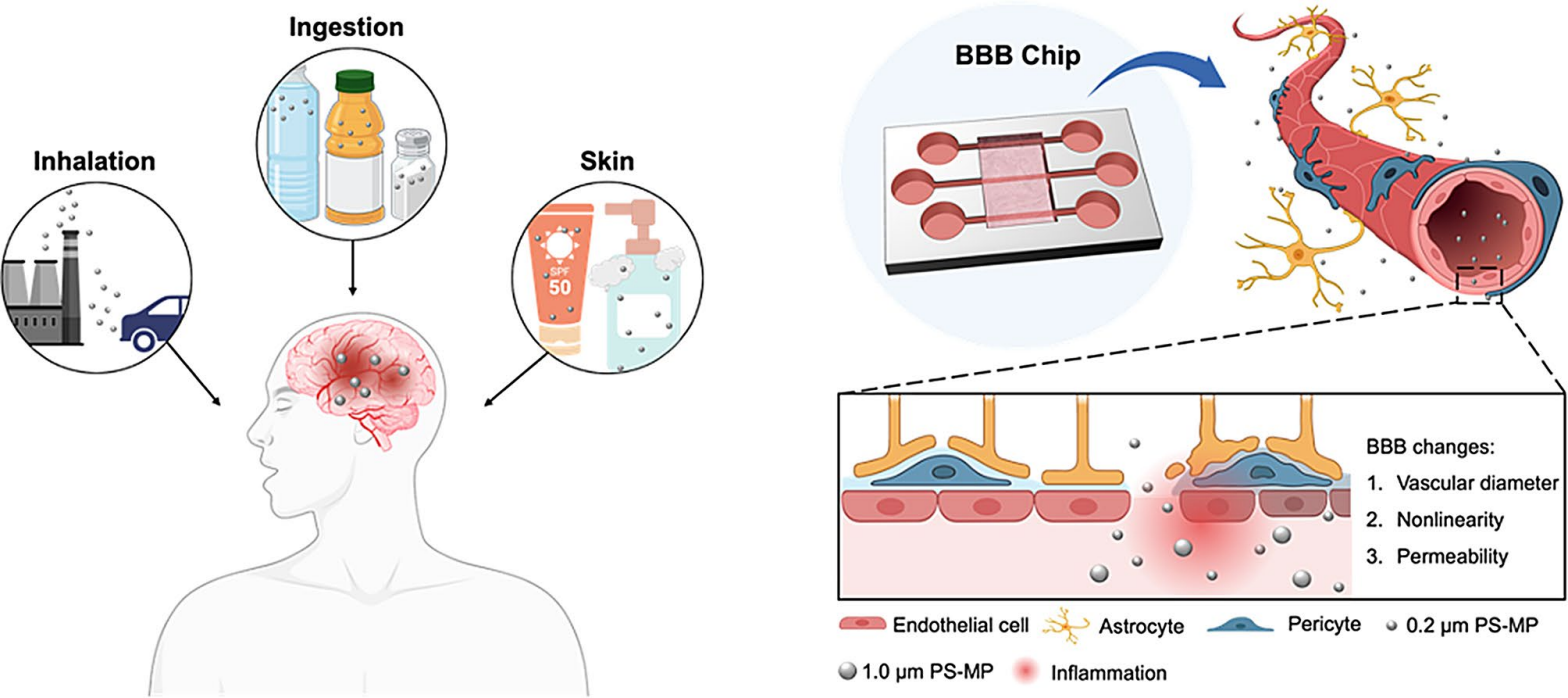

## Introduction

Plastics are polymers that can be molded under pressure, mainly made by combining monomers extracted from petroleum sources [1]. Common types of plastics include polystyrene (PS), polyethylene (PE), polyethylene terephthalate (PET), polypropylene (PP), and polyamide [2, 3]. Due to their low cost, lightweight, and durability, plastics are extensively used in everyday products such as packaging materials and containers [4]. Once released into the environment, plastic waste undergoes physical and chemical weathering, breaking down into microplastics [5]. Microplastics are plastic particles smaller than 5 mm, and nanoplastics range in size from 1 nm to 1000 nm [6]. Microplastics are classified into primary and secondary microplastics. Primary microplastics commonly include microbeads found in personal care products like facial cleansers, toothpaste, body wash, and cosmetics [7]. In contrast, secondary microplastics result from the breakdown of larger plastic items during the use or disposal through artificial actions, natural weathering, or wear [8]. Although waste microplastics initially lack functional groups or charges when they first appear through weathering process, they tend to acquire functional groups or charges over time as they interact with the environment [9–11]. Due to their diverse chemical structures and physicochemical surface properties, the modified forms of microplastics also vary widely in size, shape, chemical composition, surface charge, and reactivity [12–14]. Previous studies have confirmed that weathered plastic fragments typically exhibit a range of particle sizes and surface functional groups [15]. According to classical colloidal theories, these factors—particle size and surface groups (which influence surface charge)—are crucial in determining the reactivity, stability, and mobility of nanoparticles [16]. In this study, we utilized an amine-modified polystyrene microsphere to demonstrate their transport across BBB when they circulating though the bloodstream.

In recent years, the production of plastic products has been increasing steadily, with projections indicating that production will double within the next 20 years [17]. Additionally, the use of disposable sanitary products containing plastics, such as masks and gloves, has surged following the COVID-19 pandemic [18]. According to 2022 statics from Organisation for Economic Co-operation and Development (OECD), only 9% of plastics are recycled annually, with the majority of the remaining plastic waste ending up in the oceans. Microplastics pose significant threats to both ecosystems and human health. Due to their small size, microplastics can infiltrate various habitats, ranging from wilderness areas to densely populated urban areas, raising concerns about potential ecological impact [14]. Moreover, the pervasive use of microplastics and their ongoing environmental presence have heightened concerns about their potential risks to ecology and human health [19].

Humans are highly likely to be exposed to microplastics through the consumption of contaminated food, inhalation of airborne microplastics, and skin contact with microplastic particles contained in products and dust [20]. Microplastic particles are indigestible, and aggregates, which may contain biomolecules, can cause gastrointestinal motility disorders or blockages [21]. Previous researches have shown that the accumulation of microplastics in various tissues of aquatic animals, including the intestinal organs, liver, and brain, can induce inflammation, reproductive issues, and abnormal behaviors [22–24]. Accumulated microplastics have been shown to disrupt normal cell function, causing oxidative and inflammatory stress in cells [25]. Additionally, microplastics accumulating in the brains of mice have been found to activate microglial cells, leading to the activation of nuclear factor kappa-light-chain-enhancer of activated B cells (NF-κB), pro-inflammatory cytokines, and apoptosis markers, resulting in neuronal damage [26]. For the microplastics to enter brain tissue, they must first cross the BBB. The BBB is composed of a cerebral microvasculature made up of endothelial cells, astrocytes, and perivascular cells [27, 28]. It acts as a gatekeeper, preventing the entry of alien substances into brain tissue. However, how the mechanism by which microplastics traverse the BBB remains largely unknown.

The penetration of nano- or microparticles through BBB is particularly challenging because the BBB, along with the surrounding brain immune system, actively prevent their entry. For example, the BBB itself is highly selective in its transport properties [29], and even penetrated particles are often cleared via efflux transporters, such as P-glycoprotein [30, 31]. Additionally, particles successfully cross the BBB are quickly eliminated by immune systems. Due to these challenges, despite significant efforts to use nano- and microparticles are drug delivery carrier, they have demonstrated limited effectiveness in BBB penetration and drug delivery [32]. Nano- and micron-sized particles can enter brain tissue through various pathways, such as direct entry via intranasal route and penetration through BBB [33–36]. In this study, we aimed to replicate the transport of microparticles across the BBB during bloodstream circulation.

In this study, the effects of polystyrene microplastics on the BBB were investigated using engineered BBB models. Polystyrene beads of 0.2 μm and 1.0 μm sizes were introduced through the vascular structures of BBB. The uptake and transport of microplastics in BBB were evaluated based on the size of polystyrene particles by using microscope. The toxicity of these particles was assessed according to the size, concentration, and the duration of exposure as indicated by changes of transendothelial permeability. Additionally, the impact of inflammatory condition on the increased uptake and toxicity of microplastics in BBB was examined. It is anticipated that the engineered BBB model could serve as a valuable tool for evaluating environmental toxicity [37].

## Methods

### Fabrication of the microfluidic device

A microfluidic device was fabricated using a custom-designed metal mold with microneedles inserted. The prepolymer of polydimethyl siloxane (PDMS) (SYLGARD 184, Dow Corning, MI, USA) was mixed with a curing agent in a 10:1 (v/v) ratio. To remove microbubbles from the PDMS mixture, it was degassed in a vacuum chamber for 30 min. Subsequently, the mixture was poured into a metal mold that had been pre-inserted with three 235 μm stainless steel microneedles (Dasan Precision, SUS PIPE 32G, South Korea). To ensure a flat surface, the metal mold filled with PDMS was sandwiched between two thick glass plates and cured at 80 °C overnight. After curing, the glass plates and microneedles were removed from the PDMS, and the mold was gently separated. A collagen chamber was created using a rectangular punch measuring 10 mm in width and 5 mm in length. Holes with a diameter of 1 mm were punched in the upper right and lower left corners to create openings for collagen injection. A flat PDMS layer was bonded to the punched PDMS using oxygen plasma (Femto Science Co., South Korea). Next, six reservoirs were created using an 8 mm punch. After the microneedles were reinserted into the PDMS channels, they were treated with oxygen plasma and bonded to a cover glass. The fabricated chip was sterilized by exposing it to ultraviolet (UV) light for 20 min before the experiment. To prevent collagen detachment from the PDMS surface, a solution composed of deionized (DI) water, 10 mg/mL dopamine hydrochloride, and 100 mM pH 8.5 Tris-HCl (Sigma-Aldrich, T5931, USA) in a 7:2:1 ratio was applied to the collagen chamber. This process was conducted at room temperature on a clean bench for three hours. The surface-coated collagen chamber was washed more than three times with phosphate buffered saline (PBS; Corning, 21-040-CM, USA).

### Collagen preparation for 3D cell culture

Rat tail collagen type I (Corning, 354249, USA) was purchased and mixed with 10x Dulbecco’s Modified Eagle’s Medium (10x DMEM; Sigma-Aldrich, D2429, USA), 1x Dulbecco’s Modified Eagle’s Medium (1x DMEM; WELGENE, LM01232605, South Korea), and 1 N sodium hydroxide (NaOH; Sigma-Aldrich, 221465, USA) to create a collagen gel at a concentration of 3 mg/mL. The entire process was conducted on ice.

### Construction of in vitro vasculature within the cell-embedded collagen

Human astrocyte (HA) and human brain vascular pericyte (HBVP) were mixed at a density of 1 × 10^5^ cells/mL and embedded in the collagen gel. The collagen gel was allowed to undergo gelation by incubating it for 30 min. Subsequently, the microneedles were removed, resulting in the formation of hollow cylindrical channels of the same size as the microneedles. In order to stabilize the HA and HBVP cells, human brain microvascular endothelial cells (HBMECs) were seeded the following day. After removing all the medium from the chip reservoirs, HBMEC cells were seeded at a concentration of 3 × 10^6^ cells/mL and incubated for 15 min. A co-culture medium, consisting of a 1:1:1 mixture of HA, HBVP, and HBMEC media, was provided. During the cultivation period, the medium in the chip was replaced daily.

### Cell culture

HBMECs were purchased from ScienCell (San Diego, CA, USA) and Cell Systems (Kirkland, WA, USA). HAs and HBVPs were purchased from ScienCell (San Diego, CA, USA). All cells were cultured following the manufacturer’s recommended protocols and maintained in a humidified incubator at 37 °C with 5% CO_2_.

### Estimation of cell viability

To assess cellular toxicity, the Ez-Cytox Cell Viability Assay Kit (DoGenBio, EZ500, South Korea) was used. HA, HBVP, and HBMEC cells were seeded at a density of 0.01 × 10^6^ cells per well in a 96-well microplate and subsequently exposed to microplastics of 0.2 μm and 1.0 μm sizes at various concentrations (0, 10, 50, 100, and 200 µg/mL). After 24 and 72 h of treatment, Ez-Cytox solution was added to each well and incubated for 60 min. Absorbance was measured at 450 nm using a microplate reader.

### Polystyrene microplastic treatment in engineered BBB models

Amine-modified microspheres, 0.2 μm (Invitrogen, F8762, USA), and amine-modified polystyrene, 1.0 μm (Sigma-Aldrich, L2778, USA), were purchased. Detailed specifications regarding the microplastics used in the experiments are available in the manufacturer’s instruction manual. To deliver the microplastics through the lumen of brain microvasculature, all media were removed from the chip reservoirs. Microplastics were diluted in co-culture medium at concentrations of 10 and 100 µg/mL and administered solely to the central right reservoir. The imbalance of volume between the center right and center left reservoirs induces gravity-driven flow from right to left. The upper right and lower right reservoirs were treated co-culture medium without microplastics. It was cultured in an incubator for 30 min. After confirming the delivery of microplastics, the medium in the chip’s reservoir was removed. Subsequently, the co-culture medium was replenished, followed by incubation periods of 24 and 72 h, respectively.

### Characterization of microplastics

A clear image of the microplastics was obtained using a scanning electron microscope (SEM; Teneo VS SEM, FEI company, USA). The microplastics were air-dried on a bench overnight and then affixed to a holder using carbon tape. They were subsequently coated via an ion sputter coater E-1045 (Hitachi, Japan). The size distribution and zeta potential of each microplastic sample were analyzed using dynamic light scattering (DLS; Zetasizer Pro, Malvern, UK).

### Measurement of vascular permeability

To measure the vascular permeability of the fabricated brain endothelium, 40 kDa fluorescein isothiocyanate dextran (FITC-dextran; Sigma-Aldrich, FD40, USA) was diluted to 10 µM in phosphate buffered saline (PBS) and utilized. Molecular transport was monitored using a Zeiss LSM700 confocal microscope (Zeiss, Oberkochen, Germany), which captured sequential fluorescence images. Fluorescence images were acquired at one-minute intervals over a period of six minutes, and the time-dependent emergence of fluorescence intensity of the images was analyzed using custom-written MATLAB (MathWorks, Massachusetts, USA) codes.

### Immunofluorescence staining

The chip was fixed with 4% paraformaldehyde (Biosesang, PC2031-050-00, South Korea) for 20 minutes. Samples were then permeabilized for one hour with a blocking buffer made of PBS with 0.3% Triton X-100 (Sigma-Aldrich, T9284, USA) and 5% bovine serum albumin (BSA; Sigma-Aldrich, A2153, USA). The primary antibody was diluted at a 1:200 ratio in the blocking buffer and incubated overnight at 4°C. Subsequently, the chip was washed four times with PBS for 15 minutes each. The secondary antibody, diluted 1:500 in blocking buffer, was applied and incubated at room temperature for 2 hrs. The chip was then washed four times with PBS for 15 minutes each, and the nuclei were stained using a solution of 4’,6-diamidino-2-phenylindole (DAPI; Sigma-Aldrich, D9564, USA) at room temperature for 1 h. After a final PBS wash, images were acquired and analyzed using a Zeiss LSM700 confocal microscope (CLSM, Zeiss, Oberkochen, Germany). The primary antibody used was vascular endothelial cadherin (VE-cadherin) (Santa Cruz, SC-9989, USA) and the secondary antibody used was Alexa Fluor 488 goat anti-mouse IgG (Invitrogen, A11001, USA).

### TNF-α treatement

TNF-α, a key factor in inflammation-related diseases, was used to induce inflammatory conditions of the BBB model. The 50 ng/mL of TNF-α (R&D Systems, Minneapolis, USA) solution was introduced through the brain microvasculature of the BBB model and incubated for 2 h. Additional experiments such as microplastic exposure were performed after washing PBS.

### Statistical analysis

All experiments were repeated at least three times, and data analysis was conducted using Prism software (GraphPad Software, San Diego, CA, USA). Statistical comparisons of the analyzed values were performed using one-way ANOVA and Tukey’s HSD test. The threshold for statistical significance was set at *p < 0.05, **p < 0.01, ***p < 0.001, and ****p < 0.0001. Values not reaching statistical significance were denoted as ns (not significant) for p-values above 0.05.

## Results and discussion

**Fig. 1:**
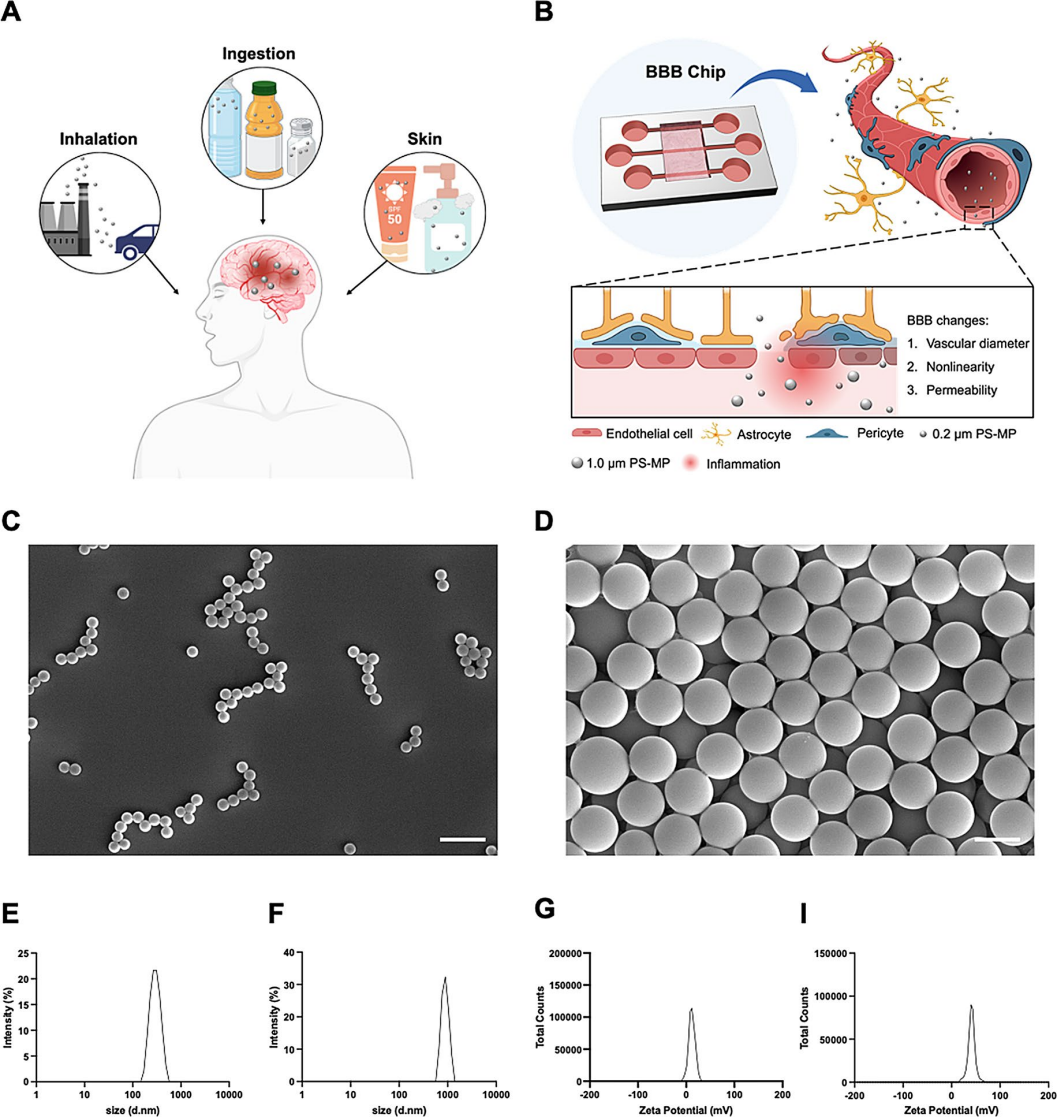
Assay of uptake and transport of microplastics by using engineered BBB models. **(A)** Schematic illustration of the pathways through which microplastics enter the human body. **(B)** Schematic illustration of the effect of PS-MPs on 3D BBB chip. Characterization of PS-MP. (C-D) SEM image of 0.2 μm **(C)** and 1.0 μm **(D)** PS-MP. Scale bar: 1 μm. **(E-F)** Hydrodynamic diameter of 0.2 μm **(E)** and 1.0 μm **(F)** PS-MPs obtained from DLS. **(G-H)** Zeta-potential analysis of 0.2 μm **(G)** and 1.0 μm **(H)** PS-MPs

Polystyrene microbeads were chosen as a model material. The morphology of the polystyrene microplastics was imaged using a scanning electron microscope (SEM), confirming that both 0.2 μm and 1.0 μm microplastics possessed a regular spherical morphology (Fig. 1C and D). The size and zeta potential of the microplastics were determined using dynamic light scattering (DLS). The hydrodynamic diameter was measured after dilution in deionized (DI) water. The 0.2 μm PS-MPs had an average diameter of 282 nm, while the 1.0 μm PS-MPs had an average diameter of 1.6 μm (Fig. 1E and F). The 0.2 μm PS-MPs had an average zeta potential of + 11.68 mV, and the 1.0 μm PS-MPs had a zeta potential of + 40.95 mV (Fig. 1G and H). As both were purchased as amine-modified polystyrene, they exhibited a positive charge.

To investigate whether microplastics elicit toxic responses in BBB constituent cells (HA, HBVP, and HBMEC), cell viability was measured using the EZ-Cytox cell viability assay kit. PS-MPs of 0.2 μm and 1.0 μm sizes were applied to 2D cultured cells at concentrations of 10, 50, 100, and 200 µg/mL. The cells were exposed to microplastics and the microplastics were not intensionally washed except the regular media changes. Media without the microplastics were changed every 24 h for cultivation. After microplastic exposure, the cells were further cultured for 24 and 72 h, respectively, to observe the uptake and cellular behaviors.

**Fig. 2:**
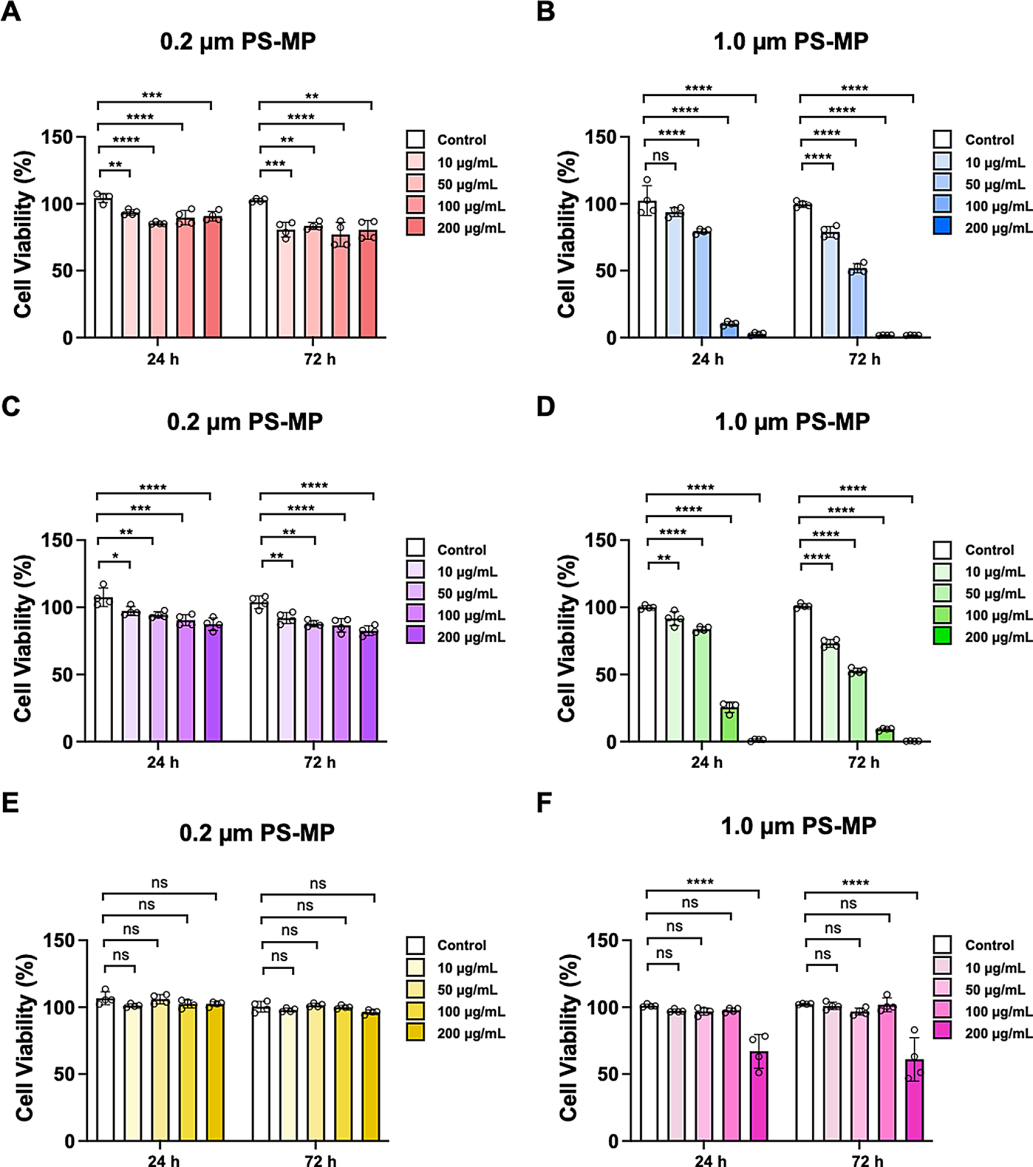
Cell viability measurements in HA, HBVP, and HBMEC cells. **(A-B)** Cell viability measurements in HA cells after 24 h and 72 h of incubation with 0.2 μm and 1.0 μm PS-MPs. **(C-D)** Cell viability measurements in HBVP cells after 24 h and 72 h of incubation with 0.2 μm and 1.0 μm PS-MPs. **(E-F) **Cell viability measurements in HBMEC cells after 24 h and 72 h of incubation with 0.2 μm and 1.0 μm PS-MPs. Data are presented as means $\:\pm\:\:SD\:$($\:n=4$ for each condition). Non-significant values are represented as ns. Statistical significance is indicated as *p < 0.05, **p < 0.01, ***p < 0.001, and ****p < 0.0001 vs. control

In HA cells, exposure to 0.2 μm PS-MPs for both 24 and 72 h led to a statistically significant reduction in cell viability at all concentrations. Similarly, exposure to 1.0 μm PS-MPs for 24 and 72 h resulted in a dose-dependent decrease in cell viability, similar to that observed with 0.2 μm PS-MPs (Fig. 2A and B). Notably, the decrease in viability was more pronounced with the larger 1.0 μm PS-MPs compared to the smaller 0.2 μm PS-MPs.

In HBVP cells, treatment with 0.2 μm and 1.0 μm PS-MPs for 24 and 72 h resulted in a decrease in cell viability at all concentrations compared to the control (Fig. 2C and D). Similar to HA cells, lower cell viability was observed with 1.0 μm PS-MPs exposure compared to 0.2 μm PS-MPs exposure.

Lastly, in HBMEC cells, no significant difference in cell viability was observed compared to the control when treated with 0.2 μm PS-MPs for 24 and 72 h. However, treatment with 1.0 μm PS-MPs showed a decrease in cell viability only at the highest concentration of 200 µg/mL (Fig. 2E and F). These results confirm that the cytotoxicity of microplastics is different depending on the cell type, size, and additional culture time.

The uptake of PS-MPs by the BBB was evaluated using engineered BBB models. The brain microvasculature in the BBB models has a tubular shape and is surrounded by HA and HBVP cells [38]. When the BBB models reached DIV 4, 0.2 μm and 1.0 μm PS-MPs were introduced through the brain microvasculature at concentrations of 10 and 100 µg/mL under flow conditions and then maintained in an incubator for 30 min for microplastic-brain microvasculature interaction. The microplastics were not intentionally washed, but the media were changed every 24 h. The microplastic-exposed BBB models were incubated for additional periods of 24 and 72 h. To investigate changes in BBB function induced by microplastics in the engineered BBB models, the expression of VE-cadherin was evaluated using immunofluorescence staining. VE-cadherin is a protein essential for maintaining adhesion among endothelial cells and the integrity of vascular structures [39].

**Fig. 3:**
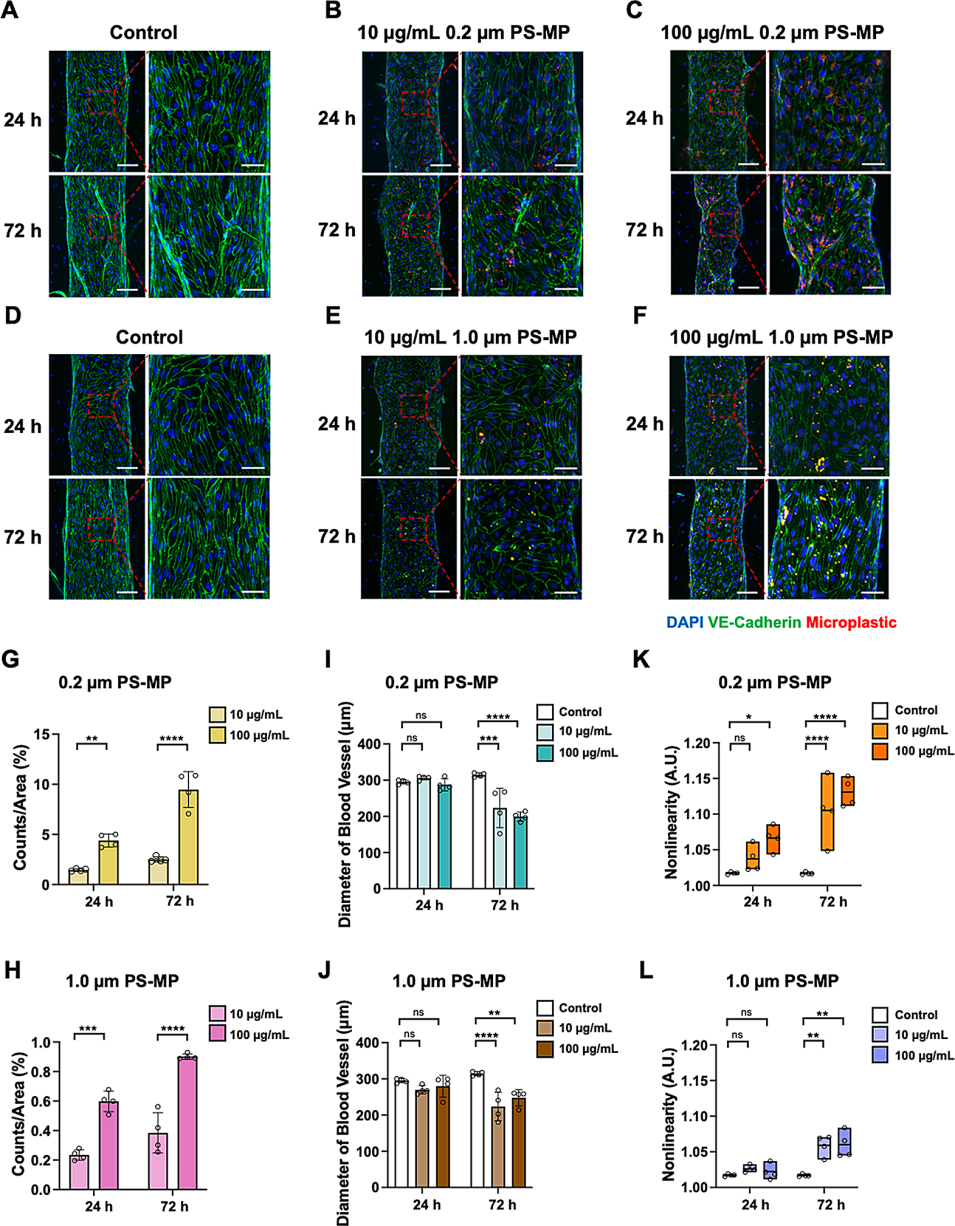
Uptake of microplastics in the brain microvasculature and morphological changes of the BBB. **(A-C)** Immunofluorescence images of 0.2 μm PS-MP-treated vasculature, showing differences based on the additional incubation time and concentration. **(D-F)** Immunofluorescence images of 1.0 μm PS-MP-treated vasculature, showing differences based on the additional incubation time and concentration. Scale bar: 100 μm (left) and 50 μm (right). **(G-H)** Accumulation analysis according to size, concentration, and additional incubation time. **(I-J)** Analysis of blood vessel diameter according to size, concentration and additional incubation time. **(K-L)** Nonlinearity analysis according to size, concentration and additional incubation time. Data are shown as means $\:\pm\:\:SD\:$($\:n=\:$4 for each condition). Non-significant values are represented as ns. Statistical significance is indicated as *p < 0.05, **p < 0.01, ***p < 0.001, and ****p < 0.0001

For visualization, 0.2 μm and 1.0 μm PS-MPs were stained red, VE-cadherin was stained green, and nuclei were stained blue (Fig. 3A-F). The polystyrene beads were more internalized into the brain endothelial cells in the higher concentrations (Fig. 3A-F). Furthermore, more microplastics were absorbed after 72 h of incubation compared to 24 h, except for the 10 µg/mL 1.0 μm particle case (Fig. 3E and F). Comparatively, it was observed that absorption increased when treated with the smaller-sized 0.2 μm PS-MPs (Fig. 3B and C). Microplastics were predominantly located around the nuclei (Fig. 3A-F).

Quantitative analysis was conducted to evaluate the damage induced by the absorbed microplastics on the BBB. First, the absorption of microplastics was quantified. The uptake of microplastics in brain endothelium increased with both the concentration and incubation time increase for both 0.2 μm and 1.0 μm PS-MPs. Comparing the absorbed amount based on the size, the smaller 0.2 μm PS-MPs were absorbed in greater quantities (Fig. 3G and H).

We utilized the diameter of brain microvasculature in the BBB model as an indicator of cellular stimulation. In this study, we focused on the long-term morphological changes after the microplastic exposure. Briefly, 72 h of incubation induced more dramatic changes in brain microvasculature diameter. For example, there was no significant difference in diameter compared to the control after 24 h of exposure to both 0.2 μm and 1.0 μm PS-MPs. However, after 72 h of incubation, a reduction in diameter was observed compared to the control (Fig. 3I and H). The reduction in diameter was more significant with the smaller 0.2 μm particles than with the larger 1.0 μm particles.

Finally, we quantified the nonlinearity (or skewness) of microvasculature since the microplastic-exposed microvasculature shows distorted morphology. The nonlinearity was quantified by dividing the trace length of the vascular walls by the straight-line length of the vessels.

$$\:Nonlinearity=\:\frac{trace\:length\:of\:vascular\:wall\:}{straight-line\:length\:of\:the\:vessel}$$

As a result, a significant difference was observed at a high concentration of 100 µg/mL after 24 h of additional incubation time with 0.2 μm PS-MPs. No significant morphological changes were observed after 24 h of incubation with the 1.0 μm PS-MPs. However, with the prolonged incubation, treatment with 0.2 μm microplastics for 72 h showed that higher concentrations resulted in increased distortion of the vascular structures. Similarly, 1.0 μm microplastics exhibited morphological changes in microvasculature after 72 h of additional incubation (Fig. 3K and L). These results indicate that the internalized microplastics induce morphological changes with a time delay. We speculate that these morphological changes are linked to the functional alterations in BBB barrier function.

The uptake of microplastics in brain endothelial cells may affect the barrier function of BBB. Tight junctions (TJs) formed between adjacent endothelial cells in brain capillaries physically restrict the entry of molecules and protect brain tissue from potential toxic substances [40]. To assess the barrier function of the BBB structure, we measured the transendothelial permeability of the fluorescent model molecule, FITC-dextran, by scanning the temporal evolution of molecular transport from brain blood vessel into the surrounding collagen space. The fluorescence around the vascular area was analyzed using a custom MATLAB code.

**Fig. 4:**
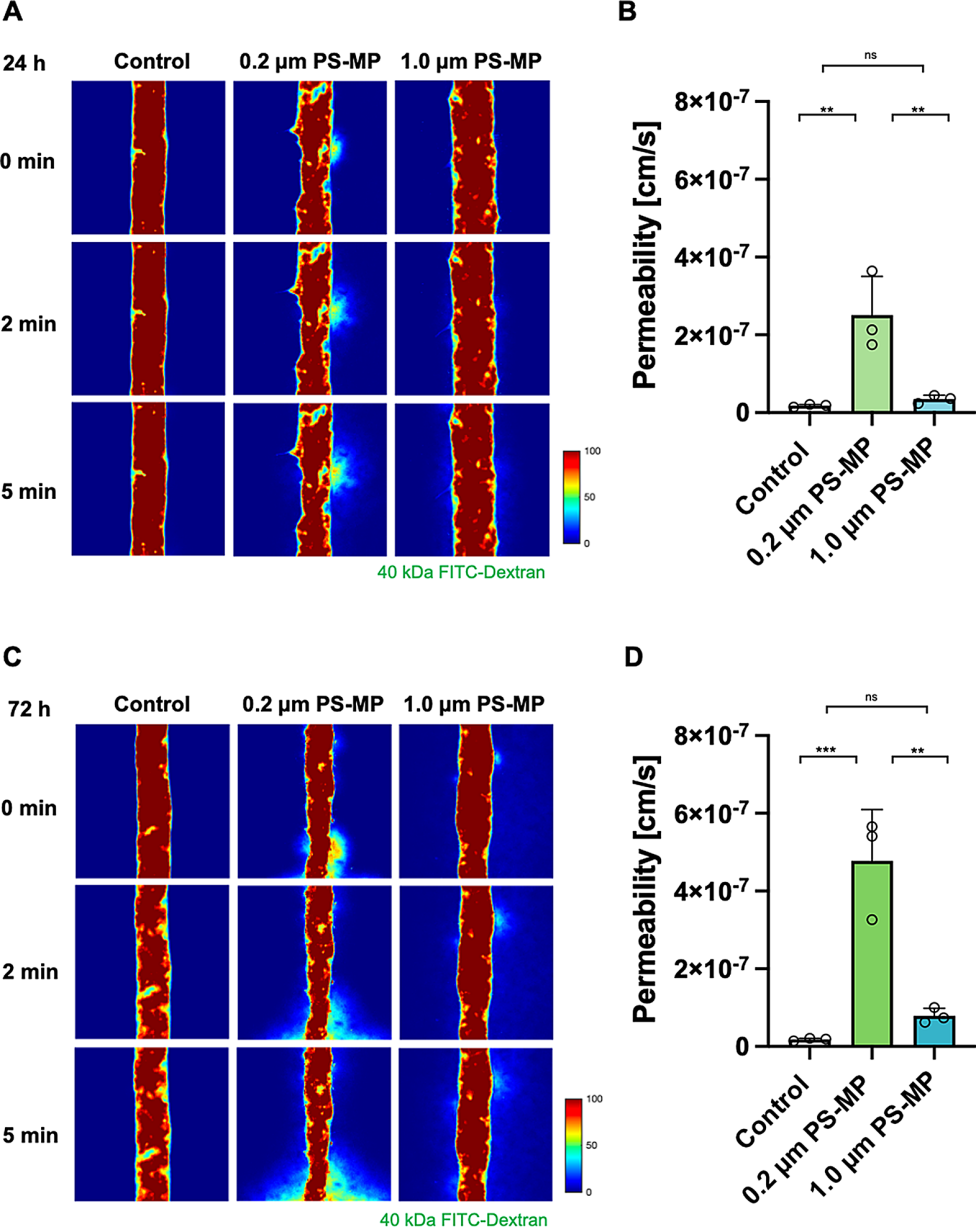
PS-MPs increase BBB permeability depending on size and additional incubation duration after exposure. **(A)** Images of 40 kDa FITC-dextran transmission through brain microvasculature after an additional 24 h of incubation following exposure to 0.2 μm and 1.0 μm PS-MPs. **(B)** Transendothelial permeability calculated based on images in **(A)**. **(C)** Images of 40 kDa FITC-dextran transmission through brain microvasculature after an additional 72 h of incubation following exposure to 0.2 μm and 1.0 μm PS-MPs. **(D)** Transendothelial permeability calculated based on images in **(C)**. Data are presented as means $\:\pm\:\:SD\:$($\:n=3$ for each condition). Non-significant values are represented as ns. Statistical significance is indicated as **p < 0.01 and ***p < 0.001

After 24 h of additional incubation following the 0.2 μm PS-MPs exposure, a significant increase in vascular permeability was observed compared to the control. Conversely, for 1.0 μm PS-MPs exposure with 24 h of additional incubation, the difference was not statistically significant (*P*_control_ = 1.735 × 10^8^ ± 1.884 × 10^9^, P_(0.2 μm)_ = 2.507 × 10^7^ ± 5.769 ×10 ^8^, P_(1.0 μm)_ = 3.476 × 10^8^ ± 5.978 × 10^9^) (Figs. 4A and B). After 72 h of additional incubation, the permeability further increased with 0.2 μm PS-MPs exposure, presumably due to the stimulation by uptaken microplastics. In contrast, 1.0 μm PS-MPs did not demonstrate a statistically significant difference in permeability (*P*_control_ = 1.735 × 10^8^ ± 1.881 × 10^9^, P_(0.2 μm)_ = 4.777 × 10^7^ ± 7.614 ×10 ^8^, P_(1.0 μm)_ = 7.884 × 10^8^ ± 1.111 × 10^9^) (Fig. 4C and D). These results demonstrate that (i) smaller microplastic sizes are associated with an increase in vascular permeability and (ii) the duration of uptake (additional incubation at this study) is a critical factor in estimating functional changes in cells. Importantly, cellular damage in 2D (shown as cell viability) was notable with larger particles (1.0 μm), while the increase in transendothelial permeability in the BBB models (a representative phenotype of disrupted barrier function) was evident in smaller particles (0.2 μm), indicating a discrepancy in cellular damage depending on the test platforms.

We hypothesized that the uptake of PS-MPs might increase under inflammatory conditions, making patients with brain diseases more susceptible to PS-MP exposure. TNF-α is a pro-inflammatory cytokine that plays a crucial role in inflammatory responses, mediating inflammation, proliferation, and cytotoxic effects in endothelial cells and almost all other cell types [41]. Additionally, this cytokine induces organizational changes in the actin cytoskeleton and the breakdown of adhesion junctions, leading to increased permeability [42]. It is generally used as a model substance to induce inflammatory responses in cells [43]. Specifically, when brain endothelial cells of the BBB are exposed to inflammatory stimuli such as TNF-α, disruption of intercellular connections occurs, leading to the breakdown of the BBB [44].

Therefore, we investigated the impact of polystyrene microplastics on the inflamed BBB. Based on previous studies, the concentration of TNF-α was set at 50 ng/mL. After treating the fabricated in vitro BBB chip with a medium diluted to 50 ng/mL of TNF-α for 2 h, 0.2 μm and 1.0 μm microplastics were introduced through the brain endothelium at a concentration of 100 µg/mL. Subsequently, the medium was replaced with co-culture medium after 30 min, and the chip was incubated additionally for 24 and 72 h.

**Fig. 5:**
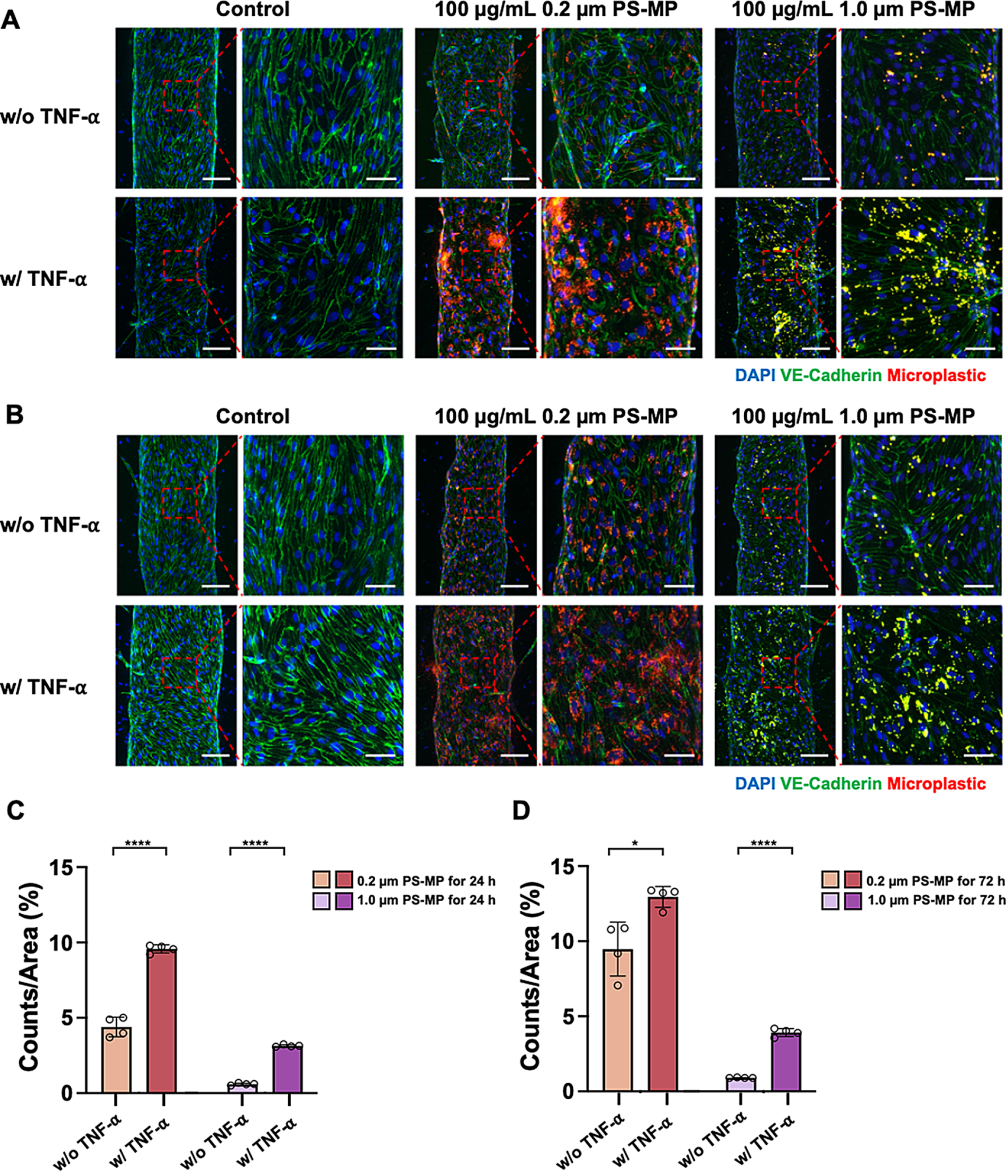
TNF-α treatment enhances PS-MP uptake in the BBB. **(A)** Immunofluorescence images of the BBB treated with 0.2 μm and 1.0 μm PS-MPs after TNF-α treatment and subsequent 24 h of incubation. **(B)** Immunofluorescence images of the BBB treated with 0.2 μm and 1.0 μm PS-MPs after TNF-α treatment and subsequent 72 h of incubation. Scale bar: 100 μm (left) and 50 μm (right). **(C)** Quantified uptake of particles per area based on images in **(A)**. **(D)** Quantified uptake of particles per area based on images in **(B)**. Data are shown as means $\:\pm\:\:SD\:$($\:n=4$ for each condition). Statistical significance is indicated as *p < 0.05 and ****p < 0.0001

To determine the extent of microplastic absorption in the inflamed BBB, immunofluorescence staining was conducted. The results indicated that the absorption of microplastics was greater after TNF-α treatment compared to non-exposed case (Fig. 5A-D). Greater absorption of microplastics in the BBB was observed when treated with 0.2 μm PS-MPs compared to 1.0 μm PS-MPs. It was also noted that the smaller (0.2 μm) microplastics were predominantly located near the nuclei of brain endothelial cells compared to larger (1.0 μm) microplastics (Fig. 5A and B). Additionally, in the absence of microplastic exposure, VE-cadherin expression increased after 72 h of incubation compared to the 24 h, suggesting that the barrier function of BBB has been restored [45]. Therefore, it was observed that the inflamed BBB, induced by TNF-α, absorbed more microplastics compared to a healthy BBB.

**Fig. 6:**
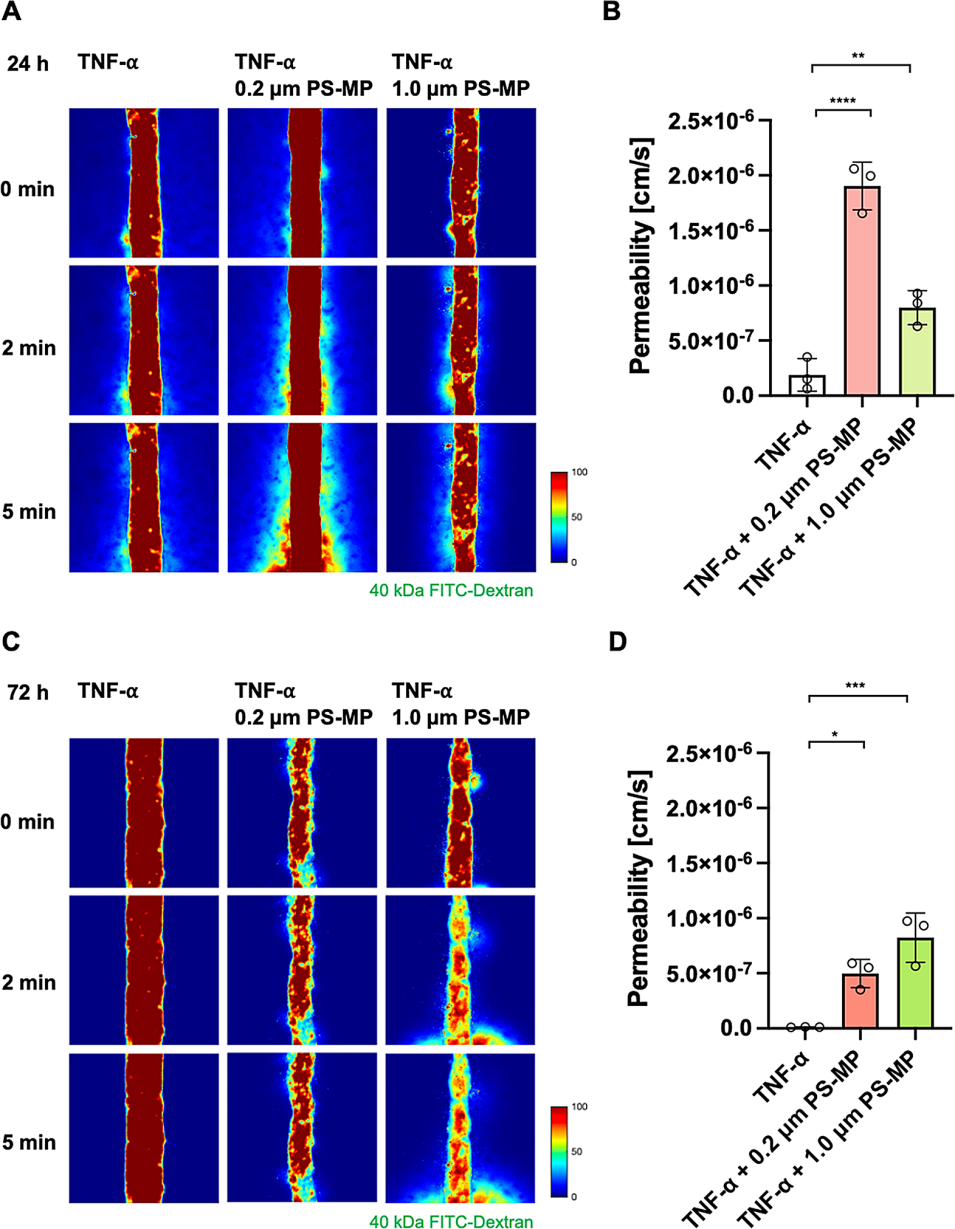
Increased BBB permeability after TNF-α treatment and subsequent microplastic exposure. **(A)** Images of 40 kDa FITC-dextran transmission through brain microvasculature after TNF-α and microplastic (0.2 μm and 1.0 μm) exposure, followed by an additional 24 h of incubation. **(B)** Transendothelial permeability calculated based on images in **(A)**. **(C)** Images of 40 kDa FITC-dextran transmission through brain microvasculature after TNF-α and microplastic (0.2 μm and 1.0 μm) exposure, followed by an additional 72 h of incubation. **(D)** Transendothelial permeability calculated based on images in **(C)**. Data are shown as means $\:\pm\:\:SD\:$($\:n=3$ for each condition). Statistical significance is indicated as *p < 0.05, **p < 0.01, ***p < 0.001, and ****p < 0.0001

After treating the 3D human brain microvascular with TNF-α, exposure to microplastics, and subsequent incubation, long-term changes in permeability were measured to observe the prolonged effects of microplastics on the BBB. Permeability was assessed using a 40 kDa FITC-dextran fluorescent solution. After TNF-α treatment followed by 24 h of incubation with microplastics, a significant increase in vascular permeability was observed for both 0.2 μm and 1.0 μm sizes compared to the control (*P*_control_ = 1.880 × 10^7^ ± 8.544 × 10^8^, P_(0.2 μm)_ = 1.903 × 10^6^ ± 1.254 ×10 ^7^, P_(1.0 μm)_ = 7.995 × 10^7^ ± 8.876 × 10^8^) (Figs. 6A and B). Compared to untreated conditions (Fig. 4), treatment with TNF-α resulted in an increased trend in BBB permeability after 24 h compared to the control, both for 0.2 μm and 1.0 μm microplastics (Fig. 6A and C). Notably, the increase in permeability was more evident in smaller (0.2 μm) microparticles. This suggests that TNF-α induced inflammatory responses, further damaging the adhesion junctions of the BBB, resulting in increased leakage of fluorescent materials.

After 72 h of incubation, a significant increase in vascular permeability was observed for both 0.2 μm and 1.0 μm microplastics compared to the control (*P*_control_ = 1.127 × 10^8^ ± 7.324 × 10^10^, P_(0.2 μm)_ = 4.976 × 10^7^ ± 7.415 ×10 ^8^, P_(1.0 μm)_ = 8.237 × 10^7^ ± 5.978 × 10^7^) (Figs. 6C and D). The recovery of vascular permeability was more notable in 0.2 μm microplastics compared to 1.0 μm microplastics. For example, after 72 h of incubation, a decrease in permeability (i.e., functional recovery) was observed for 0.2 μm microplastics compared to 24 h of incubation (*P*_*24 h 0.2 μm PS - MP*_ = 1.903 × 10^6^ ± 1.254 × 10^7^, P_*72 h 0.2 μm PS - MP*_ = 4.976 × 10^7^ ± 1.254 ×10 ^8^) (Figs. 6B and D). This suggests that the daily replacement of the medium removed remaining TNF-α, and the inherent regenerative capabilities of the brain microvascular cells partially restored the barrier function of the microvasculature. However, for 1.0 μm microplastics, the permeability did not decrease or show signs of recovery after 72 h of incubation compared to 24 h. This suggests that the size of microplastics may affect the functional recovery of BBB after the temporal stimulation [46]. Unlike cancer or infected cells, which undergo direct apoptosis in response to TNF-α, research indicates that normal cells, such as the HBMEC cells used in this study, do not directly undergo apoptosis rather exhibiting resistance to toxic stimulation [45]. Instead, the HBMEC cells displayed their damaged state through functional changes in barrier function.

In this study, we evaluated the uptake and long-term effects of polystyrene microparticles using engineered BBB models. This BBB model has a lumen structure, allowing microplastics to be administered through the brain microvasculature. It is composed of human brain-originated primary cells and closely mimics the shape and function of the human BBB. Therefore, it is believed that the uptake and toxicity of microplastics can be effectively evaluated using this human BBB-mimetic platform.

In our experiment, we assessed cellular viability in 2D culture condition using a conventional exposure protocol (treatment and maintenance) and viability assay kit. We also measured transendothelial permeability in 3D BBB models. The reason for thus dual approach is that cells in 3D BBB models did not show notable cell death even in the same concentration of microplastics, whereas cells in 2D culture exhibited significant cell death. Although the readouts are different, we believe that viability (in 2D) and transendothelial permeability (in 3D) reflect similar aspects of cellular damage. There are a few notable differences in 2D- and 3D-based experiments.

First, the size-dependent toxicity differed between 2D and 3D conditions. In 2D conditions, the cell death was more notable with larger (1.0 μm) particles than smaller (0.2 μm) particles. In contrast, in 3D BBB models, the cellular damage, indicated by increased transendothelial permeability, was more pronounced with smaller (0.2 μm) particles, showing an opposite trend compared to 2D experiment. There may be various reasons for this discrepancy, but we believe it stem from the conventional protocol, which allows excessive exposure and uptake of particles in monolayer cells.

Second, the cellular damage is temporal and may either recover or worsen over time. As shown in our microplastic exposure results, permeability further increased after 72 h of incubation compared to 24 h, presumably due to prolonged inflammatory response induced by uptaken microplastics. In the step-by-step exposure of TNF-α and microplastics, the 0.2 μm microplastic exposure case showed recovery behavior, while the 1.0 μm microplastic exposure case exhibited limited recovery of barrier function. Additionally, microplastic had much milder effects compared to diesel exhaust particle (DEP) exposure, in which brain endothelial cells exhibited significant cell death upon exposure [47]. Therefore, it apprears that the microplastics, specifically 0.2 and 1.0 μm polystyrene microbead used in this study, do not directly kill BBB-composing cells but rather deteriorate barrier function.

Despite the findings made of our study, there are several limitations when translating these results to human applications. The particles used in this study were limited to amine-modified polystyrene spheres. However, microplastics that may impact human health possess diverse characteristics, necessitating further research on microplastics with different properties, including size, charge, surface chemistry, and material composition. For example, previous studies have shown that the surface characteristics of the particles can significantly influence cellular uptake and toxicity [48–50]. In terms of particle shape, we used round-shaped particles, but many microplastics encountered in real-world scenarios exhibit irregular shapes. It is essential to investigate how these variable geometries might affect penetration behavior and toxicity in a BBB model. To comprehensively understand and mitigate the potential health risks posed by microplastics, further studies must explore not only the diversity of polymeric materials and surface characteristics but also the underlying mechanisms that drive their biological effects.

## Conclusions

In this study, we used 3D human BBB models incorporating HBMEC, HA, and HBVP to investigate the uptake and toxicity of polystyrene microplastics. We demonstrated that PS-MPs can accumulate within the BBB, causing damage and increasing permeability. The toxicity differed between 2D culture and 3D BBB models. We found that smaller-sized PS-MPs exhibited stronger toxicity in 3D BBB model, whereas larger-sized PS-MPs showed higher toxicity in 2D cultured cells. In inflamed conditions, PS-MPs caused more pronounced damage and uptake in the inflamed BBB models. Collectively, these results suggest that factors such as culture dimension and inflammation should be considered when assessing the toxicity of PS-MPs. It is believed that the 3D BBB model may serve as an effective platform for evaluating environmental toxicity.

## Data Availability

The data that support the findings of this study are available on request from the corresponding author.
